# Supplementary material for: Phylosymbiosis in the Rhizosphere Microbiome Extends to Nitrogen Cycle Functional Potential
Source: Microorganisms. 2021 Nov 30;9(12):2476. doi: 10.3390/microorganisms9122476 (PMC8709245; doi:10.3390/microorganisms9122476)
Supplement: Supplementary file 1 [file microorganisms-09-02476-s001.zip › microorganisms-1456884-supplementary.pdf]

microorganisms

MDPI

Supplementary Materials

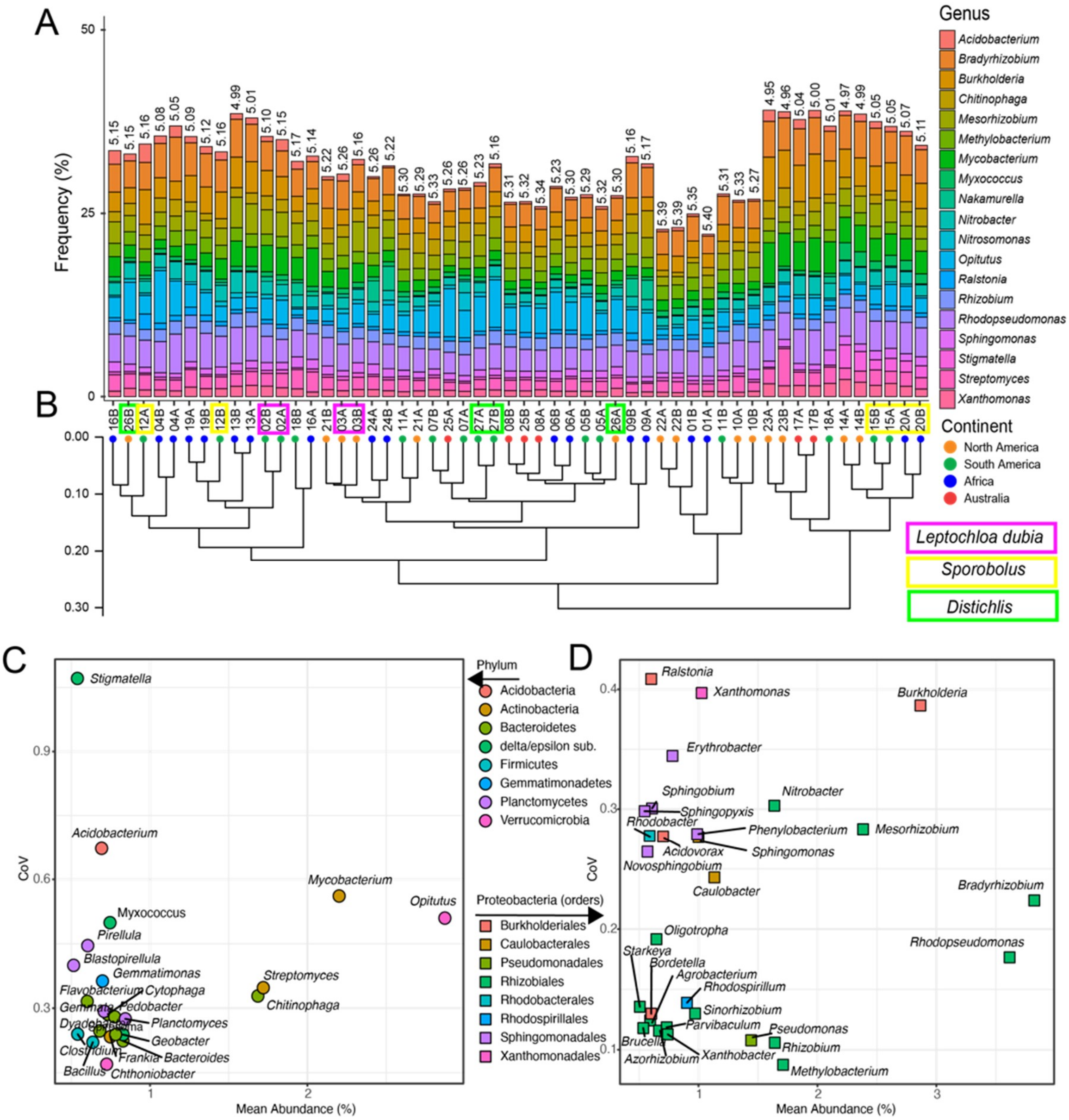

Figure S1. (A) Bar plot highlighting the most abundant genera (i.e., >2%) identified across samples. The displayed (on top of bars) Shannon diversity index (*H'*) was computed using all the taxonomically identified reads (at the genus level) for each sample, after rarefaction. (B) Microbiome clustering using all of the taxonomically annotated reads (at the genus level), using Bray-Curtis dissimilarity index and single linkage. Sample IDs (e.g., 01A) refer to the plant species (see Figure 1B, Table S1) and the sample replicate (A or B). (C, D) Coefficient of variation (CoV) of the most abundant lineages (i.e., >0.5% of the annotated reads) across samples. (C)The most abundant genera colored by phylum (without Proteobacteria). (D) The most abundant Proteobacteria colored by order.

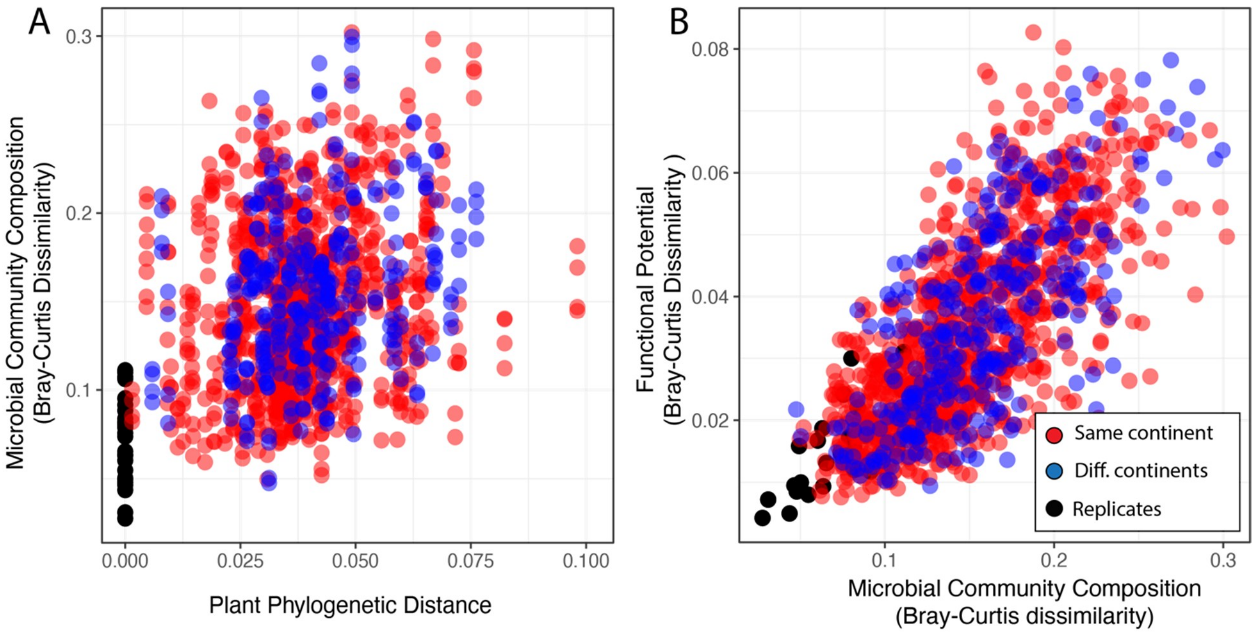

Figure S2. (A) Relation between phylogenetic distance among pairs of plants and the microbial community dissimilarity and (B) variation in the functional potential for N-cycling in the corresponding microbial communities. Comparison between technical replicates (same plant, black circle), and between samples from the same or distinct continent are highlighted.

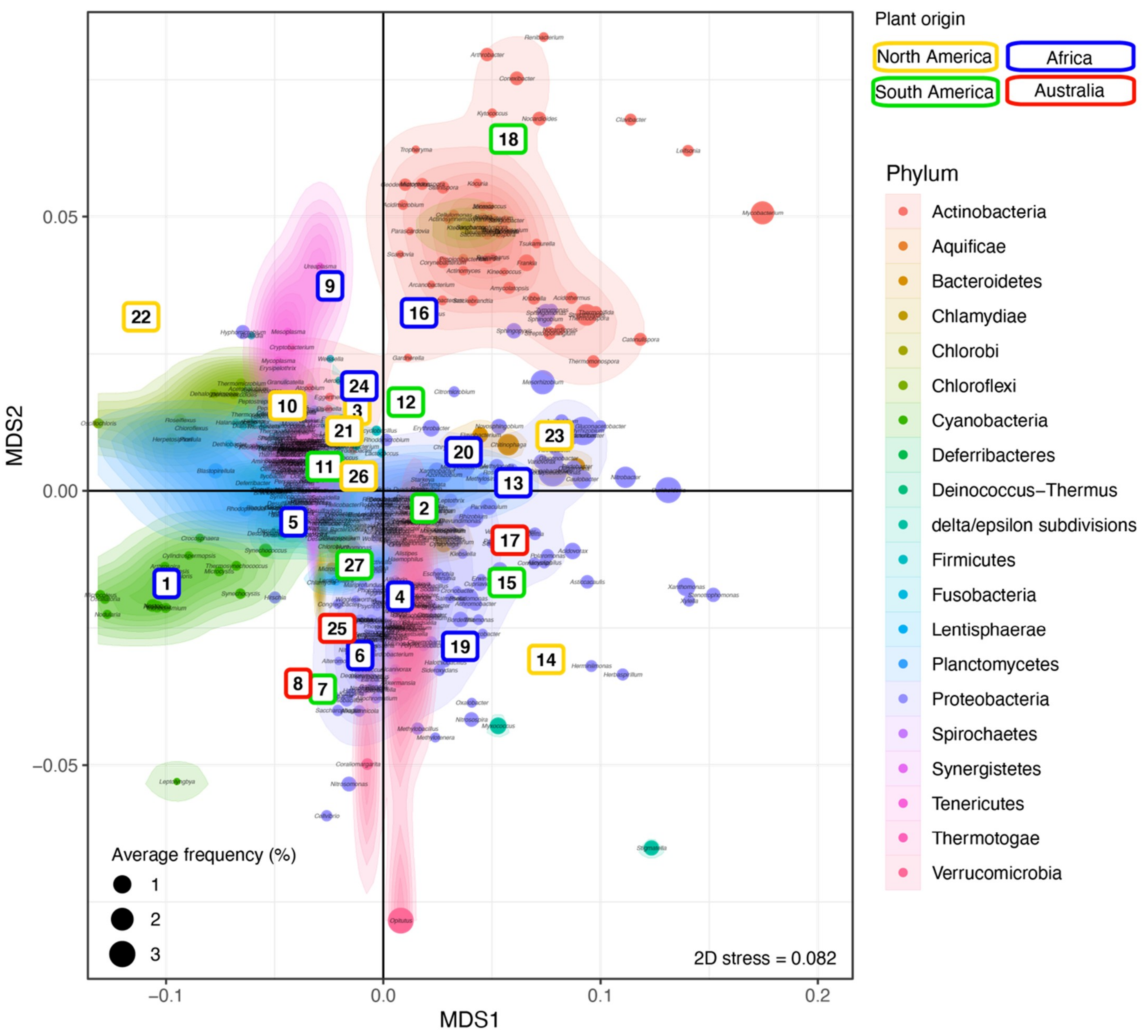

Figure S3. High resolution NMDS with all the bacterial genera identified. Microbial community structure across plants. NMDS ordination of the grass samples (colored by continent) according to their microbial community composition. The identified bacterial genera are colored by phylum and overlaid with a 2D-Kernel density plot to highlights the phyla. The genera most affected by the plant phylogeny are labeled.

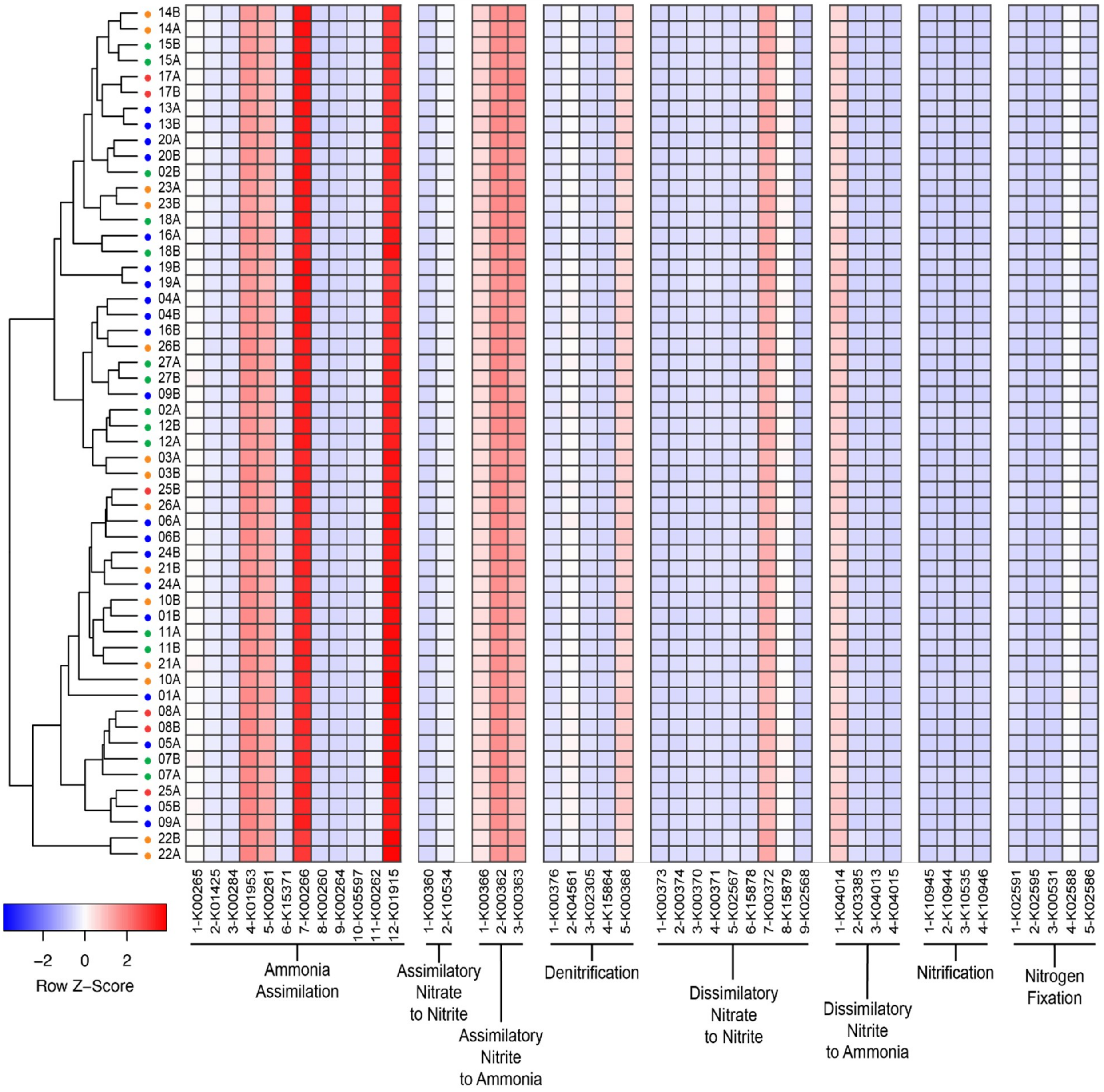

Figure S4. Functional potential for N-cycling pathways across sequenced microbiomes. The microbiomes are identified by the plant number (see Figure 1, Table S1) and replicate (A or B, see Table S2). The traits involved in N-cycling are grouped by pathways and are identified by the reaction number and KO id (see Table S3). The Z-score normalization is by row to highlight variation in N-cycling traits within samples (see Figure 4 for Z-score normalization by column to highlight variation in N-cycling traits across samples). The clustering is based on Bray-Curtis dissimilarity index and single linkage.

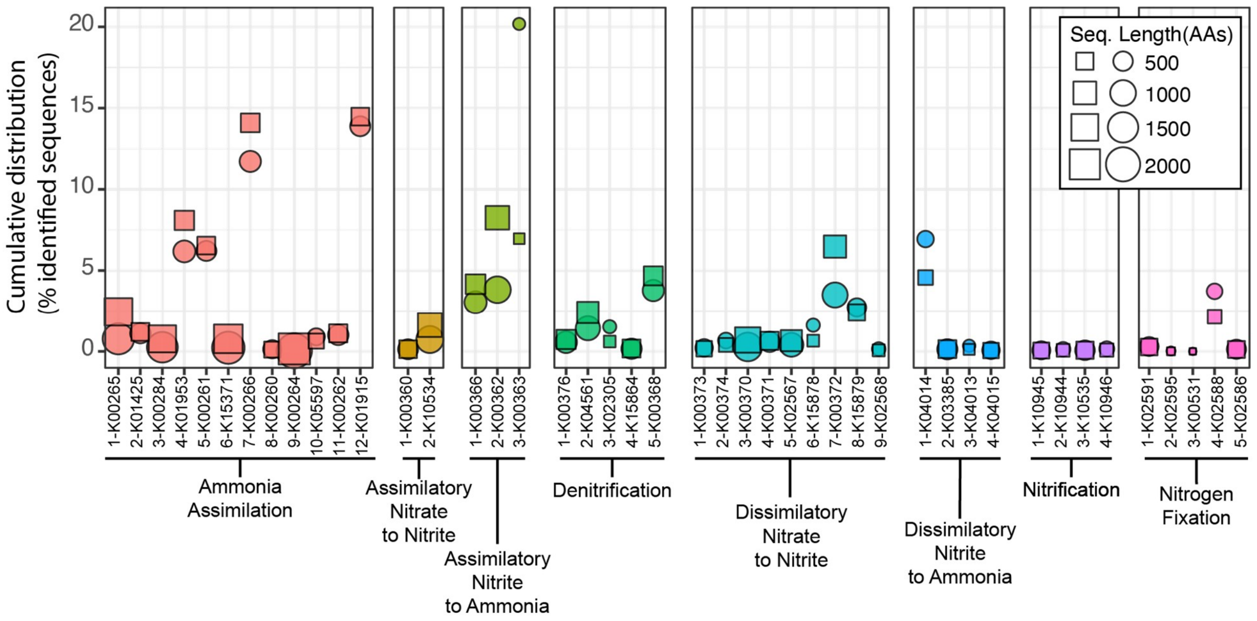

Figure S5. Cumulative distribution (%) of sequences for the N-cycling identified in the sequenced microbiomes before ( ) and after (O) accounting for the length of the targeted sequence.

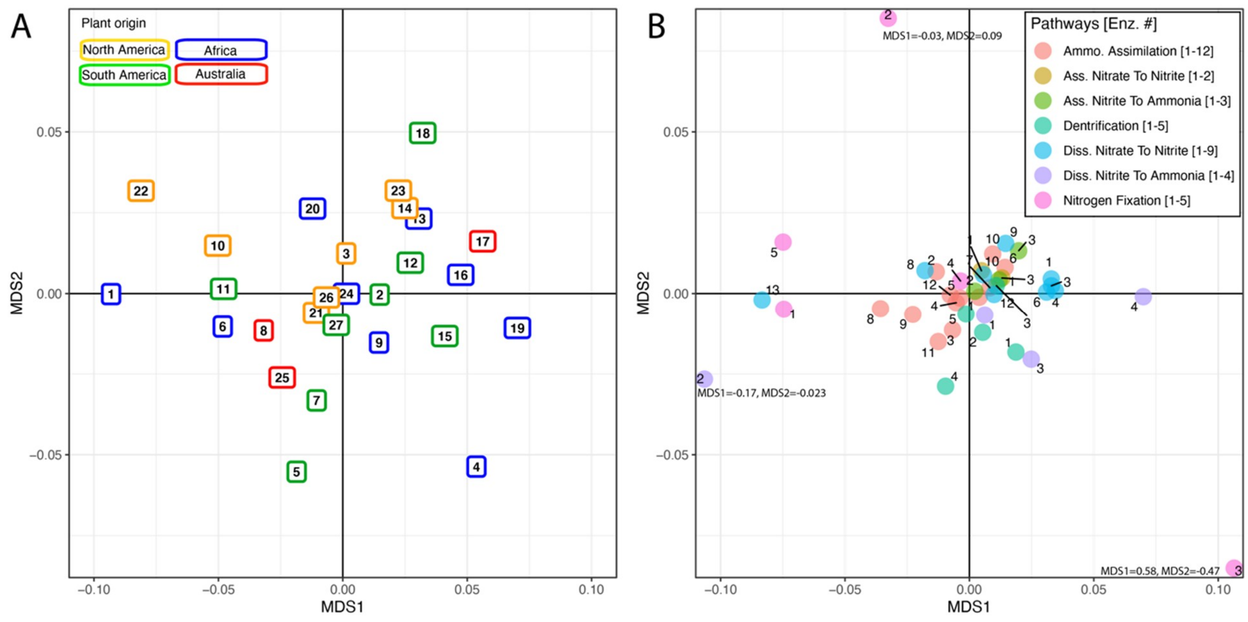

Figure S6. (A) NMDS ordination (2D stress=0.11) of the grass samples, after replicates were combined, according to (B) the rarefied distribution of reads supporting the N-cycling reactions (see Table S3).

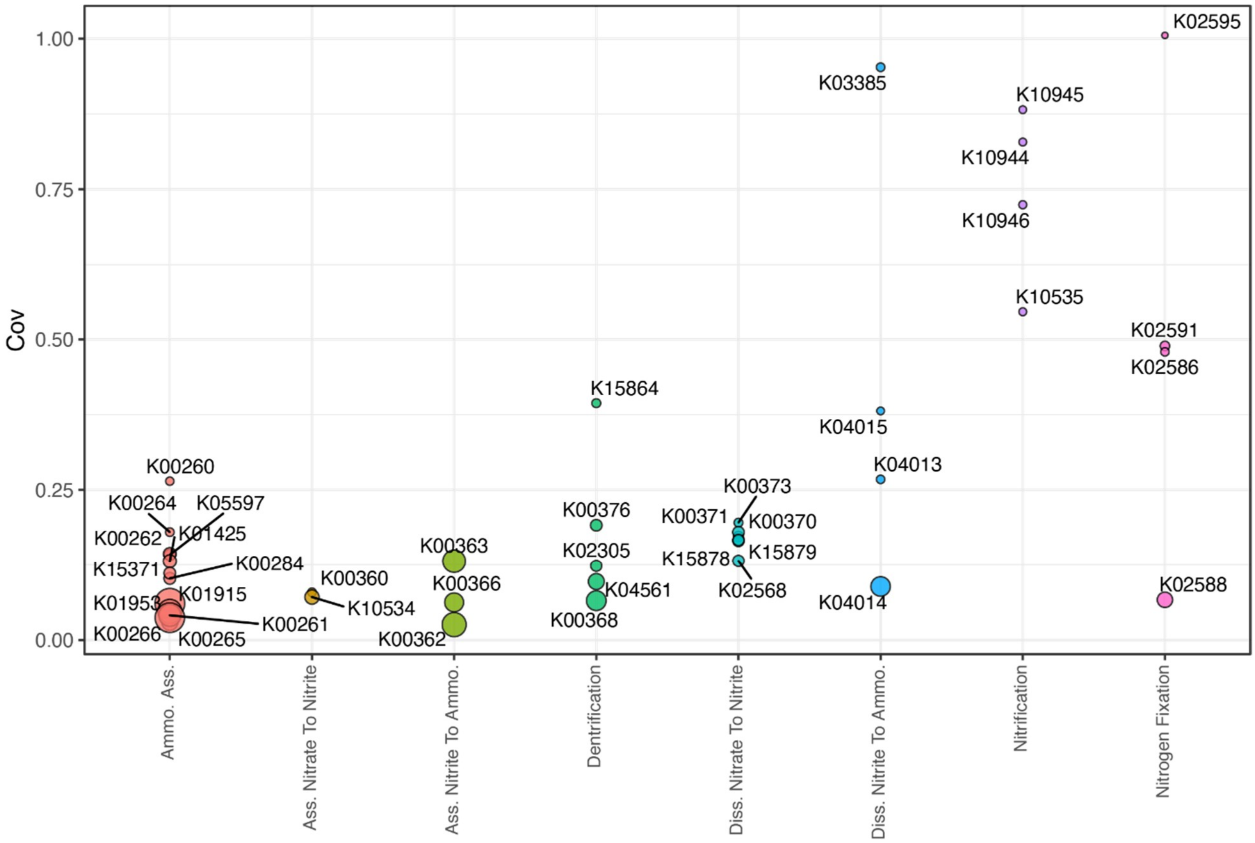

Figure S7. Coefficient of variation (CoV) of the traits supporting the N-cycling (see Table S3) across samples. Size corresponds to the relative average frequency of the trait across samples (see Figure 4A). CoV of anfG (K00531) for N-fixation pathway is not displayed.

Table S1. Grass Species Sampled with voucher specimen, Sample Origin, and GenBank Accession Numbers. P is a plastome accession number. Individual Sanger sequence accession numbers are listed in order as the *ndhF* gene, *trnC-rpoB* intergenic spacer, and *trnL-F* intergenic spacer. Plant number is used in figures. Voucher specimen indicates the collector and collection number. All vouchers are deposited at California Botanic Garden (RSA).

Plant #	Species	Voucher Specimen	Sample Origin	GenBank Accession Number
1	*Centropodia mossamadensis*	*Columbus 5680*	Namibia, Kunene Region	MW013430,MW013444,MW013458
2	*Leptochloa dubia*	*Columbus 3155*	Argentina, Prov. Catamarca	MW013433,MW013439,MW013453
3	*Leptochloa dubia*	*Columbus 2456*	USA, Arizona	P:MW014303
4	*Danthoniopsis ramosa* (outgroup)	*Columbus 5621*	Namibia, Khomas Region	P:MW014307
5*	*Odyssea paucinervis*	*Columbus 5582*	South Africa, Western Cape Prov.	P:MW014308
6	*Perotis hildebrandtii*	*Columbus 5739*	Tanzania, Pwani Region	P:MW014309
7	*Neobouteloua lophostachya*	*Columbus 3149*	Argentina, Prov. Catamarca	MW013434,MW013446,MW013459
8	Uniolinae sp.	*Simon 4500*	Australia, Queensland	P:MW014312
9	*Ctenium* cf. *concinnum*	*Columbus 5789*	Tanzania, Iringa Region	P:MW014301
10	*Hilaria cenchroides*	*Columbus 2295*	Mexico, Sonora	P:KT168387
11	*Tridens brasiliensis*	*Columbus 4816*	Argentina, Prov. Corrientes	P:MW014311
12	*Sporobolus maximus*	*Columbus 3182*	Argentina, Prov. Tucumán	P:MW014304
13	*Tetrachne dregei*	*Columbus 5491*	South Africa, Eastern Cape Prov.	P:MW014310
14	*Pappophorum vaginatum*	*Columbus 2540*	USA, Arizona	MW013435,MW013447,MW013450
15	*Sporobolus spartinus*	*Columbus 3072*	Argentina, Prov. Santa Fe	MW013436,MW013448,MW013451
16	*Triraphis andropogonoides*	*Columbus 5579*	South Africa, Eastern Cape Prov.	x,MW013442,MW013456
17	*Austrochloris dicanthioides*	*Columbus 5265*	Australia, Queensland	MW013429,MW013441,MW013455
18	*Eustachys distichophylla*	*Columbus 3090*	Argentina, Prov. Córdoba	MW013431,MW13445,MW013452
19	*Fingerhuthia sesleriformis*	*Cerros 2804*	South Africa, Western Cape Prov.	MW013432,MW013438,MW013449
20	*Sporobolus oxyphyllus*	*Columbus 5604*	South Africa, North West Prov.	MW013437,MW013443,MW013457

21	*Muhlenbergia brevigluma*	*Columbus 4772*	Mexico, Baja California Sur	x,MW013440,MW013454
22	*Eragrostis elongata*	*Columbus 3991*	USA, Hawaii, Kauai	P:MW014306
23	*Bouteloua gracilis*	*Columbus 3223*	USA, Wyoming	P:KT168392
24	*Trichoneura grandiglumis*	*Columbus 5617*	Namibia, Khomas Region	P:MW014302
25	*Astrebla pectinata*	*Columbus 5147*	Australia, Queensland	P:KT168391
26	*Distlichlis littoralis*	*Bell 544*	USA, Texas	P:MW014305
27	*Distichlis spicata*	*Bell 511*	Argentina, Prov. Santa Cruz	P:KT168395

Table S2. Metagenomes used in this study.

Plant # (table S1)	Replicate	Plant species (see table S1)	Metagenome ID	# Sequences before QC	# Sequences after QC
1	A	*Centropodia mossamadensis*	mgm4767455	28,700,675	24,085,890
1	B		mgm4851174	20,887,001	17,768,434
2	A	*Leptochloa dubia*	mgm4767419	27,265,005	23,506,585
2	B		mgm4767523	29,765,081	27,066,043
3	A	*Leptochloa dubia*	mgm4767442	28,013,016	23,893,164
3	B		mgm4767422	28,745,304	25,032,480
4	A	*Danthoniopsis ramosa*	mgm4767491	24,725,433	21,358,903
4	B		mgm4767494	29,299,148	25,280,794
5	A	*Odyssea paucinervis*	mgm4767456	25,077,269	21,744,029
5	B		mgm4767462	21,631,825	18,681,843
6	A	*Perotis hildebrandtii*	mgm4767467	26,767,811	23,096,788
6	B		mgm4767472	25,933,673	22,204,118
7	A	*Neobouteloua lophostachya*	mgm4767469	25,278,828	21,421,336
7	B		mgm4767492	22,609,724	19,607,886
8	A	Uniolinae *sp.*	mgm4767428	32,093,965	26,512,876
8	B		mgm4851173	29,111,900	25,733,484
9	A	*Ctenium* cf. *concinnum*	mgm4767452	29,881,370	25,404,466
9	B		mgm4767468	29,416,604	25,580,325
10	A	*Hilaria cenchroides*	mgm4767418	32,733,043	28,054,735
10	B		mgm4767430	30,893,383	25,413,240
11	A	*Tridens brasiliensis*	mgm4767485	27,600,940	23,672,437
11	B		mgm4767426	29,107,096	24,070,560
12	A	*Sporobolus maximus*	mgm4767448	32,531,358	28,009,579
12	B		mgm4767440	25,105,953	21,229,061
13	A	*Tetrachne dregei*	mgm4767497	27,203,650	23,840,872
13	B		mgm4767498	22,543,243	18,598,257
14	A	*Pappophorum vaginatum*	mgm4767471	26,265,997	23,133,148
14	B		mgm4767435	26,843,986	22,195,389
15	A	*Sporobolus spartinus*	mgm4767434	29,418,551	25,804,761
15	B		mgm4767450	29,471,977	23,964,915
16	A	*Triraphis andropogonoides*	mgm4767465	22,498,716	19,630,812
16	B		mgm4767487	30,875,604	25,296,555
17	A	*Austrochloris dicanthioides*	mgm4767510	23,193,679	20,521,490
17	B		mgm4767509	26,831,301	22,259,797
18	A	*Eustachys distichophylla*	mgm4767499	26,080,918	23,056,181
18	B		mgm4767484	27,416,310	22,867,877
19	A	*Fingerhuthia sesleriformis*	mgm4767477	22,484,411	19,684,461
19	B		mgm4767439	32,840,703	26,928,931
20	A	*Sporobolus oxyphyllus*	mgm4767507	31,906,851	27,869,950
20	B		mgm4767437	28,289,510	23,398,957
21	A	*Muhlenbergia brevigluma*	mgm4767483	26,534,187	23,310,600
21	B		mgm4767454	26,837,199	22,426,996
22	A	*Eragrostis elongata*	mgm4767520	27,378,506	25,427,012

22	B		mgm4767473	31,071,070	26,108,738
23	A	*Bouteloua gracilis*	mgm4767479	22,668,599	20,029,724
23	B		mgm4767417	31,037,485	26,778,054
24	A	*Trichoneura grandiglumis*	mgm4767451	22,271,002	19,499,088
24	B		mgm4767421	27,809,461	24,289,836
25	A	*Astrebla pectinata*	mgm4767490	27,878,526	23,761,008
25	B		mgm4767518	28,872,869	25,931,234
26	A	*Distlichlis littoralis*	mgm4767500	25,667,666	22,495,247
26	B		mgm4767475	31,238,182	26,638,704
27	A	*Distichlis spicata*	mgm4767493	27,263,164	23,805,480
27	B		mgm4767425	33,707,351	28,341,655
**Total**	**N/A**	**N/A**	**N/A**	**1,487,576,079**	**1,276,324,785**
**Average**	**N/A**	**N/A**	**N/A**	**27,547,705**	**23,635,644**
**SD**	**N/A**	**N/A**	**N/A**	**3,196,158**	**2,651,793**

Table S3. Reaction for N-cycling pathways identified in this study.

Pathway	React. #[a]	KO id	Domain length (AA)	Function
Ammonia Assimilation	1	K00265	1601	Glutamate synthase (NADPH) large chain [EC:1.4.1.13]
	2	K01425	546	Glutaminase [EC:3.5.1.2]
	3	K00284	1527	Glutamate synthase (ferredoxin) [EC:1.4.7.1]
	4	K01953	664	Asparagine synthase (glutamine-hydrolysing) [EC:6.3.5.4]
	5	K00261	534	Glutamate dehydrogenase (NAD(P)+) [EC:1.4.1.3]
	6	K15371	1730	Glutamate dehydrogenase [EC:1.4.1.2]
	7	K00266	610	Glutamate synthase (NADPH) small chain [EC:1.4.1.13]
	8	K00260	416	Glutamate dehydrogenase [EC:1.4.1.2]
	9	K00264	2121	Glutamate synthase (NADH) [EC:1.4.1.14]
	10	K05597	359	Glutamin-(asparagin-)ase [EC:3.5.1.38]
	11	K00262	556	Glutamate dehydrogenase (NADP+) [EC:1.4.1.4]
	12	K01915	529	Glutamine synthetase [EC:6.3.1.2]
Assimilatory Nitrate To Nitrite	1	K00360	529	Assimilatory nitrate reductase electron transfer subunit [EC:1.7.99.-]
	2	K10534	1126	Nitrate reductase (NAD(P)H) [EC:1.7.1.1 1.7.1.2 1.7.1.3]
Assimilatory Nitrite To Ammonia	1	K00366	694	Ferredoxin-nitrite reductase [EC:1.7.7.1]
	2	K00362	1096	Nitrite reductase (NADH) large subunit [EC:1.7.1.15]
	3	K00363	175	Nitrite reductase (NADH) small subunit [EC:1.7.1.15]
Dentrification	1	K00376	655	Nitrous-oxide reductase [EC:1.7.2.4]
	2	K04561	857	Nitric oxide reductase subunit B [EC:1.7.2.5]
	3	K02305	203	Nitric oxide reductase subunit C
	4	K15864	551	Nitrite reductase (NO-forming) / hydroxylamine reductase [EC:1.7.2.1 1.7.99.1]
	5	K00368	629	Nitrite reductase (NO-forming) [EC:1.7.2.1]
Dissimilatory Nitrate To Nitrite	1	K00373	384	Nitrate reductase molybdenum cofactor assembly chaperone NarJ/NarW
	2	K00374	302	Nitrate reductase gamma subunit [EC:1.7.5.1 1.7.99.-]
	3	K00370	1301	Nitrate reductase / nitrite oxidoreductase, alpha subunit [EC:1.7.5.1 1.7.99.-]
	4	K00371	560	Nitrate reductase / nitrite oxidoreductase, beta subunit [EC:1.7.5.1 1.7.99.-]
	5	K02567	824	Nitrate reductase (cytochrome) [EC:1.9.6.1]
	6	K15878	207	Rieske iron-sulfur protein
	7	K00372	941	Assimilatory nitrate reductase catalytic subunit [EC:1.7.99.-]
	8	K15879	450	Cytochrome b-561
	9	K02568	213	Nitrate reductase (cytochrome), electron transfer subunit
	1	K04014	334	Protein NrfC

Dissimilatory Nitrite To Ammonia	2	K03385	546	Nitrite reductase (cytochrome c-552) [EC:1.7.2.2]
	3	K04013	182	Cytochrome c-type protein NrfB
	4	K04015	383	Protein NrfD
Nitrification	1	K10945	413	methane/ammonia monooxygenase subunit B
	2	K10944	247	methane/ammonia monooxygenase subunit A [EC:1.14.18.3 1.14.99.39]
	3	K10535	505	hydroxylamine dehydrogenase [EC:1.7.2.6]
	4	K10946	265	methane/ammonia monooxygenase subunit C
Nitrogen Fixation	1	K02591	458	nitrogenase molybdenum-iron protein beta chain [EC:1.18.6.1]
	2	K02595	124	nitrogenase-stabilizing/protective protein
	3	K00531	117	nitrogenase delta subunit [EC:1.18.6.1]
	4	K02588	293	nitrogenase iron protein NifH
	5	K02586	477	nitrogenase molybdenum-iron protein alpha chain [EC:1.18.6.1]

[a] Nelson MB, Berlemont R, Martiny AC, Martiny JB. Nitrogen Cycling Potential of a Grassland Litter Microbial Community. *Appl Environ Microbiol*. 2015;81(20):7012-7022. doi:10.1128/AEM.02222-1.
